# *ADAM33*, a New Candidate for Psoriasis Susceptibility

**DOI:** 10.1371/journal.pone.0000906

**Published:** 2007-09-19

**Authors:** Fabienne Lesueur, Tiphaine Oudot, Simon Heath, Mario Foglio, Mark Lathrop, Jean-François Prud'homme, Judith Fischer

**Affiliations:** 1 Centre National de Génotypage, Evry, France; 2 Généthon, Evry, France; Max Planck Institute for Chemical Ecology, Germany

## Abstract

Psoriasis is a chronic skin disorder with multifactorial etiology. In a recent study, we reported results of a genome-wide scan on 46 French extended families presenting with plaque psoriasis. In addition to unambiguous linkage to the major susceptibility locus *PSORS1* on Chromosome 6p21, we provided evidence for a susceptibility locus on Chromosome 20p13. To follow up this novel psoriasis susceptibility locus we used a family-based association test (FBAT) for an association scan over the 17 Mb candidate region. A total of 85 uncorrelated SNP markers located in 65 genes of the region were initially investigated in the same set of large families used for the genome wide search, which consisted of 295 nuclear families. When positive association was obtained for a SNP, candidate genes nearby were explored more in detail using a denser set of SNPs. Thus, the gene *ADAM33* was found to be significantly associated with psoriasis in this family set (The best association was on a 3-SNP haplotype *P* = 0.00004, based on 1,000,000 permutations). This association was independent of *PSORS1*. *ADAM33* has been previously associated with asthma, which demonstrates that immune system diseases may be controlled by common susceptibility genes with general effects on dermal inflammation and immunity. The identification of *ADAM33* as a psoriasis susceptibility gene identified by positional cloning in an outbred population should provide insights into the pathogenesis and natural history of this common disease.

## Introduction

Psoriasis [MIM 177900] is a common hyperproliferative and chronic inflammatory skin disease with a prevalence of about 2–4% in Caucasian populations [1]. Plaque psoriasis, also known as psoriasis *vulgaris*, is by far the most common type of psoriasis, accounting for 80%–90% of all psoriasis patients. It appears as raised red scaling patches, most frequently on the elbows, knees, scalp and lower back.

This autoimmune disease is regarded as a multifactorial trait involving environmental factors such as intake of certain drugs, psychosocial stress, smoking, or climate conditions, all of which are well known triggering factors for primary manifestations or exacerbation in susceptible individuals [2]. On the other hand, evidence for a strong genetic component is provided by twin and family studies, which have shown a concordance rate of psoriasis in monozygotic twins of 65–72% *vs* 15–30% in dizygotic twins and a heritability of 80% [3].

In an attempt to elucidate the genetic basis of psoriasis, a number of genome-wide linkage studies have been undertaken. Overwhelming evidence for a susceptibility locus has been found for Chromosome 6p21 within the HLA region [4]–[11]. In particular, association studies employing linkage disequilibrium (LD) mapping have been successful in narrowing the locus to a 300kb interval [12]–[16] and then in identifying HLA-Cw6 as the disease allele at the 6p21 locus [16], [17]. This locus, referred as *PSORS1* (Psoriasis Susceptibility 1, [MIM 177900] contributes to the familial clustering of disease (λ) to 33<λ<50% [5], [18]. Therefore, other susceptibility genes are likely to exist. Genome-wide linkage analyses have highlighted a number of disease loci on at least 15 chromosomes (see [11] for review). Elucidation of the disease genes in these candidate loci is hampered by their large size and by the large number of candidates in each region.

We have recently confirmed the presence of a psoriasis susceptibility locus on Chromosome 20p13, through a genome-wide scan in French extended families (Peak Z_MLB_ score  =  2.9, *P*  =  0.002) [11]. This region has been previously though to be involved in the predisposition to psoriasis [4], [5] and to other inflammatory disorders such as atopic dermatitis (AD, also known as eczema) and asthma [19]. Here we follow up this finding by performing fine mapping of the 17Mb region using a family-based association study design. To maximize the chances of success, we investigated the same family set that was used for the linkage study. A total of 85 intragenic single nucleotide polymorphisms (SNPs) were initially genotyped and tested for association independently. When suggestive association was found for a marker, a denser SNP analysis was carried out to investigate the candidate genes located in the LD block. Thus, we describe the identification of *ADAM33* as being a novel psoriasis susceptibility gene. This gene encodes a member of the disintegrin and metalloprotease domain family of proteins. It has been previously associated with asthma [20]–[22], which confirms that immune system diseases are controlled by common susceptibility genes with general effects on dermal inflammation and immunity. However, this association could not be replicated in an independent set of 81 smaller families (173 nuclear families, Set II) also originating from France, indicating that different genetic factors may be involved in the predisposition to psoriasis in our population.

## Results

### Stage I: Preliminary screen for SNP-based association on Chromosome 20p13

**Figure 1:**
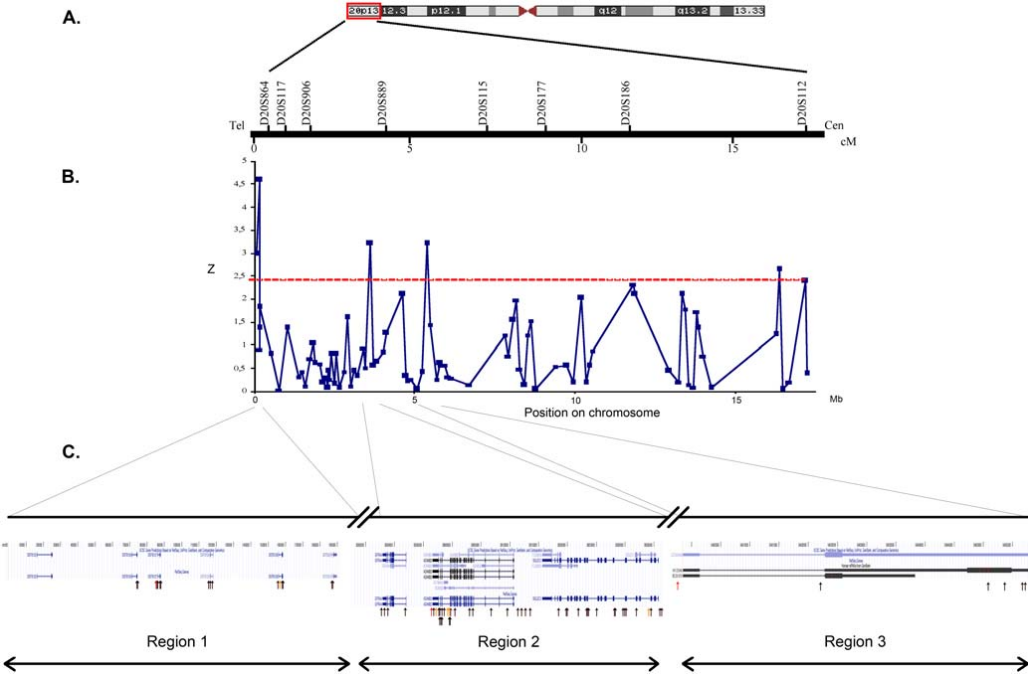
Schematic representation of the psoriasis susceptibility locus on Chromosome 20p13. A. Genetic map of the linkage interval (tel, telomere; cen, centromere). Position of microsatellites used in the linkage analysis is indicated in centimorgans (cM). B. Z plot for association tests performed with FBAT under the assumption of linkage, for SNPs selected in Stage I. Position of SNPs is indicated in megabases (Mb). The dotted line indicates the threshold for significance of the association test (a Z score>3 corresponds to a *P*<0.05). C. Detailed physical map on the contig NT_011387.8 at the 3 candidate loci. Positions of the SNPs genotyped in stage I and in stage II are shown with arrows. Red arrows indicate SNPs showing evidence for association (*P≤*0.05) in stage I; orange arrows indicate SNPs showing evidence for association (*P≤*0.05) in stage II; black arrows indicate SNPs showing no evidence for association in the univariate analysis.

The 17Mb candidate locus on 20p13 extended from the telomere of the short arm of Chromosome 20 (D20S864) to the microsatellite D20S112 (Figure 1A), and contains 428 known genes. We aimed to define the boundaries of this region of linkage and to identify the causative variants. We initially selected 85 SNPs across the region for genotyping. For all SNPs, genotypes in founders satisfied the Hardy-Weinberg equilibrium. Figure 1B illustrates the results of the family-based association test (FBAT) under the assumption of linkage [23] for the 85 SNPs genotyped in 45 multigenerational families (corresponding to 295 nuclear families). Position, minor allele frequency, and FBAT results for all SNPs are given in Supplementary Table S1. Four SNPs showed some evidence for association to psoriasis (*P*<0.05): rs12480529 located in the promoter of *DEFB127*, rs6110460 located in intron 1 of *DEFB129*, rs512625 located in the 3′UTR of *ADAM33,* and rs6053417 located in the promoter of *AK125948* encoding a hypothetical protein (Figure 1C).

### Stage II: Haplotype analyses of candidate genes at the 3 loci

We used data from the International HapMap project to look at patterns of LD surrounding the SNPs showing some association with psoriasis in the CEPH Caucasian sample set [18]. The *Defensin B* (*DEFB*) gene cluster, *ADAM33* and *AK125948* were located in 3 distinct regions (Figure 1C) and the associated markers were not in LD. To capture most of the genetic variation at the three loci, and to potentially identify allelic variant(s) predisposing to psoriasis, a tagging SNP approach was undertaken to test the candidate genes in the 3 regions (see Patients, Materials and Methods section for SNP selection). A total of 63 additional SNPs were genotyped in the same family set. Sixteen SNPs were genotyped within Region 1, 45 SNPs within Region 2 and 6 SNPs within Region 3 (Figure 1C). There was no evidence for association between the *DEFB* genes and psoriasis, nor between *AK125948* and psoriasis in the univariate SNP analyses and in the haplotypes analyses (Supplementary Table S2). However, association was observed for four additional SNPs genotyped within Region 2: three of them were *ADAM33* intronic SNPs (rs677044, rs597980, rs44707), and the fourth one (rs6076542) was in intron 3 of *SIGLEC1*, a gene located 5kb from *ADAM33*. Results of the association tests for the 45 SNPs in Region 2 are presented in Table 1.

**Table 1:** SNPs genotyped at the *ADAM33* locus and FBAT results for the univariate analysis.

SNP number	SNP ID	Other name^a^	Region^b^	Amino acid	Position^c^	Nucleotide change^d^	MAF^e^	*P* f	Database
1	rs692862		*GFRA4* IVS6+1828 bp		3578110	G>A	0.14	0.26	dbSNP
2	rs633924		*GFRA4* exon 6, 3′UTR		3580057	T>C	0.33	0.36	dbSNP
3	rs2853208		*GFRA4* exon 5	*P237P*	3580823	T>C	0.48	0.26	dbSNP
4	rs6084432		*GFRA4* IVS1+347 bp		3583653	G>A	0.16	0.93	HapMap
5	rs512625		*ADAM33* IVS22+243 bp		3588378	G>A	0.30	**0.04**	HapMap
6	rs2787094	V_4	*ADAM33* exon 22, 3′UTR		3589161	G>C	0.22	0.43	dbSNP
7	rs677044		*ADAM33* exon 22, 3′UTR		3589431	A>G	0.22	**0.03**	HapMap
8	rs543749	V_-1	*ADAM33* IVS22-33 bp		3589679	G>T	0.12	0.56	dbSNP
9	rs628977	V_-2	*ADAM33* IVS22-75 bp		3589721	C>T	0.39	0.17	dbSNP
10	rs2280089	T_+1	*ADAM33* IVS20+66 bp		3590127	G>A	0.13	0.74	HapMap
11	rs2280090	T_2	*ADAM33* exon 20	*P774S*	3590205	G>A	0.13	0.58	dbSNP
12	rs2280091	T_1	*ADAM33* exon 20	*M764T*	3590234	A>G	0.14	0.61	HapMap
13	rs574174	ST_+7	*ADAM33* IVS20-498 bp		3590694	C>T	0.18	0.35	dbSNP
14	rs597980	ST_+5	*ADAM33* IVS19+400 bp		3591165	C>T	0.45	**0.04**	dbSNP
15	rs44707	ST_+4	*ADAM33* IVS19+339 bp		3591226	A>C	0.41	**0.01**	dbSNP
16	rs598418		*ADAM33* IVS19+296 bp		3591269	T>C	0.39	0.12	dbSNP
17	rs2853209	S_+1	*ADAM33* IVS19+93 bp		3591472	T>A	0.48	0.12	dbSNP
18	rs3918396	S_1	*ADAM33* exon 19	*V710I*	3591765	G>A	0.09	0.88	dbSNP
19	rs612709	Q_-1	*ADAM33* IVS16+21 bp		3592207	G>A	0.13	0.85	dbSNP
20	rs511898	F_+1	*ADAM33* IVS6+66 bp		3595085	C>T	0.34	0.29	dbSNP
21	rs2787095		*ADAM33* IVS4-191 bp		3595943	G>C	0.39	0.21	HapMap
22	rs487377		*ADAM33* IVS2+3808 bp		3598931	G>A	0.19	0.47	dbSNP
23	rs2853213		*ADAM33* IVS1+899 bp		3601840	C>G	0.35	0.16	dbSNP
24	rs2853215		*ADAM33* IVS1-3516 bp		3606255	G>A	0.27	0.87	HapMap
25	rs535964		*ADAM33* IVS1-3747 bp		3606486	C>T	0.35	0.84	dbSNP
26	rs1046919		*ADAM33* IVS1-5086 bp		3607825	T>C	0.19	0.09	HapMap
27	rs656635		*ADAM33* IVS1-5343 bp		3608082	G>T	0.50	0.70	HapMap
28	rs17701662		*SIGLEC1* IVS18+97 bp		3610512	A>T	0.05	0.19	HapMap
29	rs4815596		*SIGLEC1* IVS17+186 bp		3611800	G>A	0.14	0.13	HapMap
30	rs3859664		*SIGLEC1* IVS17+96 bp		3611891	C>T	0.40	0.46	HapMap
31	rs3827110		*SIGLEC1* IVS13+30 bp		3614064	G>C	0.08	0.95	HapMap
32	rs3746638		*SIGLEC1* exon 11	*A974V*	3615333	A>G	0.46	0.63	HapMap
33	rs709012		*SIGLEC1* exon 11	*P919H*	3615498	G>T	0.38	0.80	HapMap
34	rs754526		*SIGLEC1* IVS11-330 bp		3615899	A>T	0.05	0.20	HapMap
35	rs525339		*SIGLEC1* IVS10+218 bp		3617014	A>G	0.38	0.47	HapMap
36	rs12624921		*SIGLEC1* IVS7-153 bp		3620259	A>G	0.05	0.29	HapMap
37	rs12624922		*SIGLEC1* IVS7-174 bp		3620280	A>G	0.05	0.20	HapMap
38	rs6139180		*SIGLEC1* IVS6+705 bp		3621283	A>G	0.11	0.72	HapMap
39	rs4813636		*SIGLEC1* IVS6+280 bp		3621708	A>G	0.37	0.46	HapMap
40	rs1018493		*SIGLEC1* exon 6	*S502S*	3622011	T>C	0.36	0.44	dbSNP
41	rs611847		*SIGLEC1* exon 5	*N350N*	3624022	C>T	0.32	0.85	HapMap
42	rs6076542		*SIGLEC1* IVS3+423 bp		3625997	T>C	0.02	**0.04**	HapMap
43	rs6037651		*SIGLEC1* exon 3	*M221V*	3626436	T>C	0.37	0.26	HapMap
44	rs735710		*SIGLEC1* IVS1-5063 bp		3632839	A>G	0.07	0.61	HapMap
45	rs4815597		*SIGLEC1* IVS1-9842 bp		3637618	C>T	0.27	0.55	HapMap

a Name according to Van Eerdewegh *et al.* [20]

b Position according to reference sequence NM_022139 for *GFRA4*, NM_025220 for *ADAM33* and NM_023068 for *SIGLEC1*

c Reference sequence: NT_011387.8, build 36 version 2 of NCBI's genome annotation

d Most common allele is given first

e Minor Allele Frequency in the studied sample set

f FBAT association test *P*-value under assumption of linkage

### Evidence for effects of combinations of *ADAM33* SNPs on the risk of psoriasis

Due to the low pairwise LD (Supplementary Table S3) and to the elevated number of haplotypes generated at the *ADAM33* locus, haplotypes covering the whole region could not be constructed. However, because multiple SNPs may act in combination to alter the risk of psoriasis, transmissions of all possible 2- or 3-SNP haplotypes to affected individuals were successively examined. The best association for the 2-SNP haplotypes was obtained for the pair SNP5/SNP23 (*P* = 0.0005, based on 1,000,000 permutations). But generally stronger associations were obtained for the 3-SNP haplotypes. The ten best 3-SNP haplotypes are presented in Table 2. Associated haplotypes involved exclusively SNPs within *ADAM33,* and revealed both risk and protective effects of *ADAM33* alleles depending on the SNP combination.

**Table 2:** Most significant under- and over-transmitted 3-SNP haplotypes for Region 2.

SNP combination^a^	Haplotype	Set I					Set II					Set I + Set II			
		Frequency %	Number of informative families^b^	Z^c^	*P*	1,000,000 Permutations *P*	Frequency %	Number of informative families^b^	Z^c^	*P*	1,000,000 Permutations *P*	Frequency %	Z^c^	*P*	1,000,000 Permutations *P*
SNP 5/10/25	AGT	10	54.6	(-)3.32	0.0009	0.00004	11	37.8	0.90	0.37	0.39	10	(-)2.27	0.02	0.01
SNP 5/11/23	AGG	11	70.5	(-)3.33	0.0009	0.0003	10	28.5	1.44	0.15	0.16	11	(-)2.10	0.04	0.03
SNP 5/25/26	ATT	10	54.1	(-)3.32	0.0009	0.00007	12	36.4	1.12	0.26	0.29	11	(-)2.12	0.03	0.02
SNP 5/26/27	ATG	9	51.2	(-)3.32	0.0009	0.001	9	20.8	0.64	0.52	0.57	9	(-)2.48	0.01	0.02
SNP 7/9/23	ACC	32	133.3	3.36	0.0008	0.001	29	46.2	0.91	0.36	0.34	31	3.25	0.001	0.002
SNP 7/16/23	ATC	32	127.7	3.31	0.0009	0.002	28	41.1	0.69	0.49	0.47	31	3.08	0.002	0.003
SNP 7/21/23	AGC	22	105.7	3.37	0.0008	0.0007	15	32.5	(-)0.004	0.99	0.96	19	3.18	0.001	0.003
SNP 15/23/24	ACG	33	125.9	3.45	0.0006	0.002	30	48.8	0.29	0.77	0.78	32	2.97	0.003	0.005
SNP 16/23/27	TCG	11	67.8	3.49	0.0005	0.001	11	18.4	(-)1.10	0.27	0.30	11	2.18	0.03	0.04
SNP 16/26/27	TCG	10	60.1	3.34	0.0008	0.0006	8	14.7	0.13	0.89	0.95	9	3.14	0.002	0.002

a SNP numbers refer to SNPs in Table 1.

b Number of informative families estimated by FBAT

c Score given by the association test on haplotypes performed by FBAT, under the assumption of linkage. A positive Z indicates an increased risk, whereas a negative Z indicates a protective effect on the expression of psoriasis.

### Association of *ADAM33* with psoriasis is independent of *HLA-Cw6* status

The 20p13 locus is likely to segregate independently of *PSORS1* in the psoriasis families [11]. However, a potential interaction between *ADAM33* and *HLA-*Cw6 alleles was tested. *HLA-Cw6* positive patients were identified using SNPs in strong LD with the risk allele (namely HCR-325 C>T (rs130076), HCR-1723 G>T (rs130079), HCR-2327 C>G (rs1576) and CDSN971 C>T (rs1062470) [24], [25]. As expected, those markers define a strongly associated haplotype in our population (*P*<0.000001 for haplotype H2, based on 1,000,000 permutations, Supplementary Table S4). Association between *ADAM33* and psoriasis was then monitored using FBAT when stratifying the families according to the presence or absence of this risk haplotype. Although the number of informative families was reduced, the associations between *ADAM33* 3-SNPs haplotypes and psoriasis were still observed in the group of patients not carrying *HLA-Cw6*, indicating that the 2 loci act independently. As expected, the associations were less significant in the group of patients carrying *HLA-Cw6*, due to the stronger contribution of the 6p21 locus to psoriasis susceptibility (Supplementary Table S5).

### Genetic heterogeneity between large, multigenerational families and smaller families segregating psoriasis

We attempted to confirm our findings of association between *ADAM33* and psoriasis in a broader family sample. Thus, a second set of 81 smaller French families was investigated (Set II). Those families had not been included in the linkage study due to the low informativity of the pedigrees for such studies. However, they represented 173 nuclear families on which the FBAT transmission disequilibrium test could be performed (Table 3). Therefore, the 15 SNPs contributing to the best combinations were genotyped in Set II (Table 4). No association was found in the individual or combined SNP analyses when analyzing Set II independently, but same 3-SNP haplotype associations were confirmed when combining Set I and Set II. It is likely that the discrepancy between the 2 family sets was due to a lower informativity of Set II because of the difference in the pedigree structures (Table 3). Indeed, the numbers of informative nuclear families for the 3-SNP haplotypes giving the best associations in Set I were lower in Set II than in Set I (Table 2). Moreover, the results of *HLA-C* tagging SNPs, known to be strongly associated with psoriasis, illustrated the weak power of Set II for performing family-based association tests. For example, for SNP rs1062470, a *P* of 0.0001 was obtained for Set I, which had 86.7% (39/45) of informative families, whereas a *P* of 0.02 was obtained for Set II but this set had only 50.6% (41/81) of informative families. However, a significant association was observed for the combined sets (*P* = 0.000006, Supplementary Table S4).

**Table 3:** Comparison of Set I and Set II.

*A. Description of pedigrees used in the association study*																											
Family set	Number families			Number of nuclear families							Number of subjects								Average number of subjects per family						Number of genotyped subjects (Affected)		Average number of genotyped subjects per family (Affected)
Set I	45			295							1161								25.80						926 (346)		20.58 (7.69)
Set II	81			173							668								8.25						539 (235)		7.32 (2.90)
*B. Distribution of pedigrees according to the number of nuclear families per pedigree*																											
Number of nuclear families per pedigree			1			2		3		4			5		6		7		8		9		10	>10		Total	
Set I			1			5		5		6			2		8		4		1		2		4	7		45	
Set II			43			8		16		10			1		2		0		0		1		0	0		81	
*C. Distribution of nuclear families according to the sibship size*																											
Number of sibships per nuclear families		1			2		3		4			5		6		7		8		9		10		14		Total	
Set I		75			91		58		31			10		8		11		5		3		2		1		295	
Set II		40			66		47		11			5		4		0		0		0		0		0		173	

**Table 4:** Univariate SNP analysis in Set II and in combined sets.

SNP number	SNP ID	Gene	Set II		Set I + Set II	
			Number of informatives families	*P* a	Number of informatives families	*P* a
5	rs512625	intergenic	32	0.19	65	0.42
7	rs677044	*ADAM33*	34	0.98	62	0.10
9	rs628977	*ADAM33*	35	0.80	69	0.22
10	rs2280089	*ADAM33*	26	0.62	54	0.96
11	rs2280090	*ADAM33*	24	0.79	52	0.79
14	rs597980	*ADAM33*	28	0.50	63	0.17
15	rs44707	*ADAM33*	35	0.60	71	0.11
16	rs598418	*ADAM33*	35	0.63	71	0.36
21	rs2787095	*ADAM33*	36	0.64	70	0.46
23	rs2853213	*ADAM33*	35	0.84	69	0.34
24	rs2853215	intergenic	30	0.73	61	0.74
25	rs535964	intergenic	32	0.82	67	0.99
26	rs1046919	intergenic	26	0.92	59	0.14
27	rs656635	intergenic	39	0.77	71	0.63
42	rs6076542	*SIGLEC1*	5	-b	15	0.53

Only SNPs showing some association with psoriasis in Set I, in the univariate or in the 3-SNP haplotype analyses, were tested in Set II.

a FBAT association test *P*-value under assumption of linkage.

b Association test could not be performed due to insufficient number of informative families.

## Discussion

A common genetic component to autoimmune susceptibility had been initially shown by twin and adoption studies and by increased risk to siblings [26]. Later, linkage studies demonstrated that autoimmune diseases share a limited number of loci [27], [28], reinforcing the idea that common susceptibility genes control them. Recently, we identified a susceptibility locus for psoriasis on Chromosome 20p13 [11], a region also linked to AD and asthma, two other inflammatory disorders [19]. Here, using a family-based association design to interrogate the locus, we identified several combinations of SNPs within *ADAM33,* a gene that has been associated with asthma in many studies, to be strongly associated with psoriasis in multigenerational French families.

Due to the large number of all possible 2- and 3-SNP combinations tested, the issue of multiple testing should be addressed here. A Bonferroni correction, although too conservative because the SNPs in the candidate region were all correlated, could be applied. When considering all possible 2-SNP combinations with one of the 5 SNPs associated in the univariate analysis, 110 tests are performed. Therefore, the threshold for a significant *P*-value should be <0.00045. The best 2-SNP combination (SNP5/SNP23) gives a *P* = 0.0005, based on 1,000,000 permutations, in favour of a significant association with psoriasis. Moreover, these 2 SNPs are also included in some of the best 3-SNP combinations (Table 2), which is again in favor of a true association.

These associations were not observed in a set of much smaller families. Although statistical type I errors in Set I cannot be totally discarded here, the discrepancy between the 2 family sets could be accounted for by a lack of informativity of Set II families. Another possible explanation could be that the selection of highly predisposed family enriched Set I for individuals carrying risk alleles at a smaller number of loci with stronger effects, whereas psoriasis susceptibility in Set II may be due to a higher number of loci with weaker effects. Thus, the contribution of specific *ADAM33* alleles to the familial clustering and individual risk prediction of psoriasis is likely to be relatively small, and these issues of case selection should be addressed in future replication studies.

Nevertheless, our findings can be the source of valuable physiological insights, since several *ADAM33* SNPs have been found to be associated with asthma and with bronchial hyper-responsiveness in Caucasians, in African Americans and in Hispanics [20]–[22]. An association of *ADAM33* with allergic rhinitis has also been reported in the Japanese population [29]. This first report of an association between *ADAM33* and psoriasis confirms that common biological pathways may be involved in the etiology of psoriasis and other clinically distinct immune-mediated diseases.

Although clinical data on inflammatory and autoimmune diseases other than psoriasis were limited in our family sample, we examined personal or family history of atopy (AD, asthma and allergic rhinitis) and of seborrheic dermatitis (SD) retrospectively from the data available from the questionnaire answered by family members (Set I). SD was reported in 34 families (75.6%, 128 subjects), AD in 25 families (55.6%, 63 subjects), asthma in 22 families (47.8%, 34 subjects), and allergic rhinitis in 5 families (10.9%, 5 subjects). These data indicate a higher incidence of chronic inflammatory diseases in the psoriasis families than in the general population, except for allergic rhinitis (Incidences of SD, AD, asthma and allergic rhinitis in France are respectively: 1–3%, 2–5%, 5–7% and 15%) and support the existence of common genes interacting with other genetic or environmental factors to result in distinct immunologic abnormalities. As in the general population, these diseases rarely occurred in the same patient in our family sample, indicating that susceptibility alleles for these disorders are likely to be different. Indeed, *ADAM33* SNPs that have been associated with asthma are not the SNPs defining protective and risk haplotypes for psoriasis [20]. The recently reported colocalization of the susceptibility loci for psoriasis (*PSORS4*) and atopic dermatitis (*ATOD2*) on Chromosome 1q21 also supports this hypothesis [30]. Although immunologic processes in psoriasis and AD are quite different and the two diseases rarely occur together in the same patient, the possibility of a specific misregulation of the *LOR* gene at 1q21, which is down-regulated in psoriasis, and up-regulated in AD has been suggested [30]. In the light of our data, the involvement of *ADAM33* should be further investigated in AD as well.

Once an allelic association with the disease has been demonstrated, the identification of causal variants is less straightforward. The *ADAM33* gene consists of 22 exons that have been re-sequenced in different populations for SNP identification [20], [31]. Of the numerous SNPs described in the public SNP databases, only 4 validated SNPs occur in the coding region of the gene and, of these, 3 are non-synonymous. We have excluded an association between two of them, T764M (rs2280091) and S774P (rs2280090), and psoriasis (Table 1). The third SNP, A178T (rs3918392), was also genotyped in our family set, but association tests could not be performed due to its low frequency (3%) in our population. In asthma studies, it has been proposed that 3′UTR polymorphisms may be significant [32], although functional investigations of some of them have so far been unsuccessful [33]. Interestingly, SNP 7 (rs677044) in the 3′UTR of *ADAM33* showed some association when analyzed on its own and was present on all 4 most significant protective haplotypes (Table 2). However, this SNP was also present on other haplotypes not associated with the disease. Therefore, a functional role of this SNP in psoriasis susceptibility should be discarded. Finally, *ADAM33* gene undergoes complex alternative splicing with several variant transcripts and their relative functional significance to each other is not clear [33], [34]. It has been suggested that some of the *ADAM33* polymorphisms may affect alternative splicing, splicing efficiency or mRNA turnover [20] but such functional effects for SNP5 (rs512625) in the 3′ region of *ADAM33* and for SNP14 (rs597980) and SNP15 (rs44707) in intron 19 of the gene were not investigated in this study.

It has already been noticed that the individual effect of a variant can be too weak to be detected individually and that interactions of multiple SNPs within the same gene can affect a phenotype [35]. In complex situations of gene involvement, Jannot *et al.* showed that testing combinations of SNPs could provide better power than testing each single SNP for association [36]. In type 2 diabetes, *CAPN10* and *NOD2* are two examples where haplotypes made up of non-coding variants have been associated with disease phenotypes in complex fashion while no association was seen in the univariate SNP analyses [37]. The same situation is observed in the case of asthma and psoriasis, where the association with *ADAM33* is stronger when combinations of SNPs are examined.

A recent study confirmed that the *ADAM33* locus shows extended linkage disequilibrium upstream of *ADAM33* to *GFRA4*, as well as downstream including *SIGLEC1* (also named sialoadhesin *SN*) [38]. The region can be divided into 5 haplotype blocks, *ADAM33* being situated between block 4 and 5, with an increased recombinatory rates around exons S to V of *ADAM33*. Half of the SNPs included in the associated combinations here lie in exon S or upstream (SNPs 5, 7, 9, 10, 11, 15, 16) and the second half lie downstream exon F (SNP 21, 23, 24, 25, 26, 27). Resolution of LD maps and block definition at *ADAM33* locus is still noisy and it is likely that yet unidentified variant(s) within the *ADAM33* gene or within distant regulatory elements may be responsible for asthma or psoriasis. Deep resequencing of the full region would be required to identify such functional relevant variation.

Psoriasis is a chronic disorder in which T-cell-mediated inflammation causes thickening of the skin. Conversely, it has been also hypothesized that in psoriatic patients, the lack of control of the outer skin cells may lead to the greatly increased production of cells that characterizes psoriasis. This, in turn, may lead to an abnormality of the blood vessels and the inflammation characteristic of psoriasis. Another possibility is that epidermal skin cells fail to mature into the flat, thickened, “cornified” layer they are supposed to. As a result, the epidermis tries to produce more cells than usual leading to the thickened epidermis, which then leads to inflammation. ADAM proteins have a complex organization that includes a signal sequence and the following domains: pro, metalloprotease (including a zinc-binding sequence), disintegrin, cysteine-rich, epidermal growth factor, transmembrane, and cytoplasmic tail domains. The proteins have diverse functions which include adhesion, cell fusion, intracellular signaling and the shedding of the extracellular portion of proteins such as cytokines and growth factors, leading to the soluble forms of these proteins. Expression data suggest that ADAM33 is expressed in most human tissues, including skin [39]. It is biologically plausible that ADAM33 is relevant to the development of psoriasis because it may be involved in the inflammatory response, or in cell-cell and cell-matrix interactions that are essential for the development and maintenance of a tissue; likewise, extracellular matrix proteolysis is an important contributor to skin remodeling, which when altered might ultimately lead to significant desquamation or, perhaps, absence of cell maturation.

To conclude with, this is the first report of an association between *ADAM33* and psoriasis. Confirmation of our findings in different populations would represent an important development in understanding susceptibility to psoriasis, allergy, and closely related phenotypes. The importance of this observation should be evaluated by further delineating the biological role of *ADAM33* in psoriasis.

## Materials and Methods

### Families

The French psoriasis study was approved by the Ethics Committee of Le Kremlin-Bicêtre Hospital in 1995 (CCPPRB) [40]. Briefly, families were identified through a media campaign between 1996 and 2001 at Généthon, using posters in the Paris Métro and information in news magazines, radio and television. Clinical diagnoses were checked by systematic telephone calls to every family member, affected or non-affected, for each family, at least twice during four years by dermatologists using a standard questionnaire. The attending physician of each patient was also contacted, usually by mail, which led to confirmation of the diagnosis in over 75% of cases. Thus, 126 families were enrolled in the genetic study and provided blood samples. These families were divided in two non-homogeneous family sets (Set I and Set II): Set I corresponded to the 45 highly predisposed multigenerational families used for the initial genome-wide scan, and included on average 8 affected members per family [11], whereas Set II corresponded to the 81 remaining smaller families (3 affected members per family on average, Table 3). One inbred large family was reported in Set I, with parents who were first cousins (inbred coefficient for children  =  1/16). DNA was extracted from whole blood using standard procedures after written informed consent of subjects. The study was conducted in accordance with the Declaration of Helsinki Principles.

### SNP selection

SNPs were initially identified through the HapMap database. A list of 402 validated SNPs located between the microsatellite markers D20S864 and D20S112 was generated. In order to perform a first scanning of the region with a limited number of SNPs, biallelic markers were filtered according to the following criteria: selected markers had a minor allele frequency (MAF)≥20% in Caucasian populations, were located within known genes or nearby (+10 kb upstream and downstream of known genes), and SNPs with ambiguous flanking sequences were excluded for genotyping. The population frequencies for the SNPs were taken from the CEU HapMap population (CEPH collection of Utah residents of northern and western European ancestry). Thus, 85 SNPs whose position was representative of the overall marker distribution were eligible for genotyping for Stage I. These SNPs were located in or near 65 different genes and were not in linkage disequilibrium with each other (1 SNP/137 kb on average).

To select additional SNPs in the 3 candidate regions on 20p13 (Stage II), different strategies were applied depending on the availability of SNP data for each candidate gene. When coverage of a gene with HapMap SNPs was sufficient, we used the Tagger program to select SNPs that efficiently tagged all common variations of the candidate genes [41]. This was the case for *SIGLEC1* and *AK12594*5: 17 HapMap tagging SNPs across *SIGLEC1* (Table 1) and five HapMap tagging SNPs across *AK12594*5 (Supplementary Table S2) were genotyped in family set I. For these two genes, two additional common SNPs that were absent in HapMap database (rs1018493, located in exon 6 of *SIGLEC1* and rs1060236, located in the untranslated region of *AK12594*5) were also genotyped.

When coverage with HapMap SNPs was insufficient, the density of markers across a candidate was increased using validated SNPs from dbSNP database or from the literature. This was the case for *ADAM33*. This gene had been resequenced in different populations [20], [31], and a number of reported SNPs were not present in the HapMap database. Therefore, in addition to 8 HapMap SNPs, 15 other SNPs were selected. Those included all 3 validated nonsynonymous SNPs (with frequency≥5% in Caucasians) and SNPs that had been previously shown to be associated with asthma either in univariate or in haplotype analyses. These SNPs included F_-1 (rs3918392), F_+1 (rs511898), Q_-1 (rs612709), S_1 (rs3918396), ST_+4 (rs44707), ST_+7 (rs574174), V_-2 (rs628977), V_-1 (rs543749) and V_4 (rs2787094) [20] (Table 1). Two additional SNPs previously found to be associated with asthma, V_2 (rs3918400) and V_5 (rs3746631), did not assay successfully; however, given the moderate to strong levels of LD of SNPs previously documented, we expected the studied SNPs across the region to capture most of the haplotypic variation.

Very few SNPs had been identified in *GFRA4,* despite the fact that the whole coding sequence of the gene had been resequenced ([42] and personal communication). Therefore, only 4 SNPs were selected for genotyping in our family set.

Finally for genes within Region 1 (*DEFB125, DEFB126, DEFB127, DEFB128, DEFB129* and *DEFB32)*, incomplete SNP data were available in the public SNP databases when this study was initiated. Therefore, we resequenced the entire coding sequence and the exon/intron junctions of the 6 *DEFB* genes in 58 unrelated Caucasian controls. Tagging SNPs were identified using the tagsnps program [43].

### SNP genotyping

Genotyping was carried out using Taqman® according to manufacturer's instructions. Primers and probes were supplied directly by Applied Biosystems as Assays-by-Design™. All assays were carried out in 384-well plates. Each plate included negative controls (with no DNA) and positive controls were duplicated on a separate quality control plate. Plates were read on the ABI PRISM 7900 using the Sequence Detection Software (Applied Biosystems, Foster City, California, United States). Failed genotypes were not repeated.

Genotypes were checked for Mendelian inheritance errors using FBAT [23] and PEDSTATS was used to discard SNPs which deviate from Hardy-Weinberg Equilibrium in unrelated subjects [44].

### Statistical analyses

Family based association analysis was carried out using the FBAT program to examine the transmission rates of marker alleles under the assumption of linkage. The FBAT test is a multiallelic test based on the classic transmission/disequilibrium test (TDT) developed by Spielman *et al.* [45]. It considers parents heterozygous for a certain allele at the marker locus associated with the disease and evaluates the frequency with which that allele is transmitted to affected offspring. In each trio, the untransmitted alleles of the parents serve as controls. The FBAT method permits analysis of family structures larger than trios. It has been shown that, when data on parents are missing, one case and two sibs bring similar power levels to trios and adding sibs when parents are available increases power [23]. The FBAT software decomposes pedigrees into individual nuclear families and treats them as independent in most of the calculations. The pedigree's contribution to the FBAT test statistics is then obtained by summing over all nuclear families within the pedigree. However, in the case where linkage is present and the null hypothesis states “linkage, but no association”, the genotypes of the different nuclear families derived from one pedigree are correlated. Even with a single nuclear family, the transmissions to multiple sibs are correlated when linkage is present. Therefore, when testing for association in an area of known linkage with multiple sibs in a family or when multiple families in pedigree occur, an empirical variance for the test statistics should be used. We used the –e option of FBAT to compute the “corrected” test statistic, and gave the *P*-value “*P*” corresponding to this corrected test statistic. Furthermore, haplotype analysis was performed using the HBAT function of FBAT, under the assumption of linkage. This is a method for estimating genetic association from probabilities of haplotype transmission to affected offspring. To circumvent the problem of multiple testing due to the large number of statistical tests performed simultaneously in the association study, the false discovering rate was controlled and permutation *P*-values were computed with FBAT program (1,000,000 permutation tests were performed).

### Electronic databases and programs

HapMap data are available at http://www.hapmap.org (public data released n° 6 at 2005-03-01). The dbSNP database is available at http://www.ncbi.nlm.nih.gov/SNP/. The University of California Santa Cruz assembly of the genome is available at http://genome.ucsc.edu/.

The FBAT program version 1.5.5 is available at http://www.biostat.harvard.edu/∼fbat/. PEDSTATS is available at http://www.sph.umich.edu/csg/abecasis/PedStats/. Tagsnps is available at http://www-rcf.usc.edu/∼stram/tagSNPs.html. Tagger on Haploview, version 3.2 is available at http://www.broad.mit.edu/mpg/haploview/using.php.

## Supporting Information

Table S1 Results of the association tests for SNPs selected in Stage I.
(0.16 MB DOC)

Table S2 Results for DEFB genes and for AK125948 gene.
(0.10 MB DOC)

Table S3 Pairwise LD (D') for the 17 SNPs genotyped in the whole family set.
(0.02 MB XLS)

Table S4 Results for HLA-Cw6 tagging SNPs (PSORS1 locus).
(0.09 MB DOC)

Table S5 Results for most significant under- and over-transmitted ADAM33 3-SNP haplotypes when stratifying according to HLA-Cw6 status in patients (for Set I).
(0.07 MB DOC)
