# Supplementary material for: ADAM33, a New Candidate for Psoriasis Susceptibility
Source: PLoS One. 2007 Sep 19;2(9):e906. doi: 10.1371/journal.pone.0000906 (PMC1975467; doi:10.1371/journal.pone.0000906)
Supplement: Table S1 — Results of the association tests for SNPs selected in Stage I. (0.16 MB DOC) [file pone.0000906.s001.doc]

**Supplementary Table S1. Results of the association tests for SNPs selected in Stage I.**

| SNP | MAF^a^ | Gene | Position^b^ | Distance from previous SNP | *P*^c^ |
| --- | --- | --- | --- | --- | --- |
| rs12480529 | 0.32 | *DEFB127* | 77454 | - | **0.05** |
| rs6110460 | 0.46 | *DEFB129* | 148168 | 70714 | **0.01** |
| rs1053783 | 0.07 | *DEFB129* | 150306 | 2138 | 0.41 |
| rs373225 | 0.37 | *DEFB32* | 178507 | 28201 | 0.25 |
| rs399083 | 0.34 | *DEFB32* | 179688 | 1181 | 0.16 |
| rs282163 | 0.17 | *TCF15* | 528089 | 348401 | 0.44 |
| rs1045410 | 0.34 | *C20orf55* | 766666 | 238577 | 0.98 |
| rs6040024 | 0.19 | *PSMF1* | 1048262 | 281596 | 0.25 |
| rs3795135 | 0.22 | *NSFL1C* | 1381841 | 333579 | 0.74 |
| rs2253429 | 0.49 | *PTPNS1L2, SIRPB1* | 1482954 | 101113 | 0.66 |
| rs6110838 | 0.42 | *SIRPB2* | 1585502 | 102548 | 0.90 |
| rs200894 | 0.36 | *LOC388779* | 1729827 | 144325 | 0.50 |
| rs1884564 | 0.13 | *PTPNS1* | 1812658 | 82831 | 0.35 |
| rs2235749 | 0.24 | *PDYN* | 1899939 | 87281 | 0.54 |
| rs6075668 | 0.50 | *STK35* | 2043264 | 143325 | 0.56 |
| rs6035842 | 0.30 | *LOC388780* | 2123608 | 80344 | 0.82 |
| rs214818 | 0.21 | *TGM3* | 2240017 | 116409 | 0.75 |
| rs6082805 | 0.46 | *TGM3* | 2267417 | 27400 | 0.92 |
| rs2422753 | 0.26 | *TGM6* | 2301684 | 34267 | 0.64 |
| rs2076649 | 0.34 | *TGM6* | 2350952 | 49268 | 0.79 |
| rs4446127 | 0.25 | *SNRPB, MGC10715* | 2397788 | 46836 | 0.44 |
| rs6050260 | 0.43 | *TMC2* | 2495157 | 97369 | 0.85 |
| rs6083931 | 0.44 | *NOL5A, TMC2* | 2565120 | 69963 | 0.44 |
| rs2073126 | 0.37 | *KIAA1442* | 2668985 | 103865 | 0.92 |
| rs600832 | 0.43 | *PTPRA* | 2814086 | 145101 | 0.66 |
| rs6084229 | 0.48 | *PTPRA* | 2918928 | 104842 | 0.20 |
| rs6084265 | 0.34 | *AVP* | 3010898 | 91970 | 0.90 |
| rs6051639 | 0.38 | *ITPA, C20orf116* | 3120283 | 109385 | 0.64 |
| rs6139068 | 0.20 | *C20orf194* | 3224393 | 104110 | 0.71 |
| rs6076524 | 0.37 | *ATRN* | 3392447 | 168054 | 0.40 |
| rs235577 | 0.22 | *ATRN* | 3481633 | 89186 | 0.61 |
| rs512625 | 0.30 | *GFRA4, ADAM33* | 3588378 | 106745 | **0.04** |
| rs2281479 | 0.23 | *CENPB, C20orf28* | 3702095 | 113717 | 0.57 |
| rs6084506 | 0.40 | *PANK2* | 3810932 | 108837 | 0.52 |
| rs4813669 | 0.38 | *MGC34919* | 4032860 | 221928 | 0.43 |
| rs6084657 | 0.42 | *SMOX* | 4111497 | 78637 | 0.28 |
| rs2756271 | 0.38 | *PRNP* | 4605262 | 493765 | 0.12 |
| rs2422982 | 0.29 | *RASSF2* | 4706197 | 100935 | 0.71 |
| rs1110277 | 0.40 | *SLC23A2* | 4794682 | 88485 | 0.80 |
| rs6139593 | 0.46 | *SLC23A2* | 4892923 | 98241 | 0.79 |
| rs4239761 | 0.16 | *CDS2, PCNA* | 5054584 | 161661 | 0.95 |
| rs6085093 | 0.44 | *LOC388785, GPR73L1* | 5242653 | 188069 | 0.65 |
| rs6053417 | 0.46 | *AK125948* | 5387617 | 144964 | **0.04** |
| rs6053519 | 0.39 | *KIAA1434* | 5506109 | 118492 | 0.24 |
| rs6107689 | 0.37 | *FLJ25067* | 5686348 | 180239 | 0.79 |
| rs1287021 | 0.22 | *FLJ25067* | 5764893 | 78545 | 0.54 |
| rs236141 | 0.37 | *CHGB* | 5831907 | 67014 | 0.58 |
| rs6085352 | 0.27 | *C20orf75* | 5944157 | 112250 | 0.58 |
| rs2295435 | 0.43 | *C20orf42* | 6036695 | 92538 | 0.74 |
| rs2259248 | 0.29 | *LOC219414* | 6128859 | 92164 | 0.76 |
| rs1005464 | 0.24 | *BMP2* | 6696148 | 567289 | 0.88 |
| rs6117980 | 0.29 | *HAO1* | 7816255 | 1120107 | 0.30 |
| rs6140479 | 0.26 | *DJ971N18.2* | 7897766 | 81511 | 0.47 |
| rs6039040 | 0.17 | *PLCB1* | 8047397 | 149631 | 0.21 |
| rs2142669 | 0.48 | *PLCB1* | 8161389 | 113992 | 0.14 |
| rs6055745 | 0.29 | *PLCB1* | 8252279 | 90890 | 0.63 |
| rs1534968 | 0.27 | *PLCB1* | 8419274 | 166995 | 0.87 |
| rs4813865 | 0.37 | *PLCB1* | 8522403 | 103129 | 0.30 |
| rs6077412 | 0.29 | *PLCB1* | 8635471 | 113068 | 0.22 |
| rs724110 | 0.17 | *PLCB1* | 8750049 | 114578 | 0.94 |
| rs1474670 | 0.35 | *PLCB4* | 9396707 | 646658 | 0.59 |
| rs926496 | 0.47 | *PAK7* | 9724204 | 327497 | 0.57 |
| rs596203 | 0.25 | *ANKRD5* | 9960229 | 236025 | 0.82 |
| rs6074113 | 0.47 | *SNAP25* | 10182011 | 221782 | 0.13 |
| rs6108571 | 0.43 | *LOC128710, MKKS* | 10350320 | 168309 | 0.82 |
| rs6040010 | 0.38 | *LOC128710* | 10451586 | 101266 | 0.57 |
| rs2179687 | 0.36 | *JAG1* | 10554295 | 102709 | 0.42 |
| rs6033250 | 0.40 | *BTBD3* | 11805731 | 1251436 | 0.10 |
| rs3177118 | 0.45 | *BTBD3* | 11845642 | 39911 | 0.12 |
| rs3843766 | 0.47 | *C20orf38* | 12921561 | 1075919 | 0.64 |
| rs4814218 | 0.60 | *C20orf82* | 13223462 | 301901 | 0.83 |
| rs743183 | 0.31 | *C20orf13* | 13337227 | 113765 | 0.12 |
| rs6042192 | 0.30 | *C20orf13* | 13436848 | 99621 | 0.17 |
| rs6109983 | 0.43 | *C20orf13* | 13552157 | 115309 | 0.88 |
| rs6042333 | 0.14 | *C20orf6* | 13667740 | 115583 | 0.93 |
| rs998182 | 0.39 | *C20orf50* | 13767653 | 99913 | 0.18 |
| rs6079226 | 0.42 | *C20orf50* | 13858640 | 90987 | 0.25 |
| rs6074678 | 0.37 | *C20orf133* | 13971392 | 112752 | 0.47 |
| rs6079395 | 0.50 | *FLRT3* | 14267899 | 296507 | 0.92 |
| rs2180443 | 0.45 | *C20orf23* | 16280836 | 2012937 | 0.29 |
| rs6111142 | 0.27 | *C20orf23* | 16384164 | 103328 | 0.07 |
| rs6135798 | 0.38 | *C20orf23* | 16485359 | 101195 | 0.94 |
| rs4813245 | 0.46 | *OTOR, SNRPB2* | 16664770 | 179411 | 0.83 |
| rs6044705 | 0.42 | *PCSK2* | 17180001 | 515231 | 0.09 |
| rs926492 | 0.29 | *PCSK2* | 17251577 | 71576 | 0.67 |

^a^Minor Allele Frequency in the studied sample set

^b^Reference sequence: NT_011387.8, build 36 version 2 of NCBI’s genome annotation

^c^FBAT association test *P*-value under the assumption of linkage
