# Supplementary material for: ADAM33, a New Candidate for Psoriasis Susceptibility
Source: PLoS One. 2007 Sep 19;2(9):e906. doi: 10.1371/journal.pone.0000906 (PMC1975467; doi:10.1371/journal.pone.0000906)
Supplement: Table S2 — Results for DEFB genes and for AK125948 gene. (0.10 MB DOC) [file pone.0000906.s002.doc]

Supplementary Table S2. Results for *DEFB* genes and for *AK125948* gene

| *A. Univariate analysis* | | | | | | | | | | | | | |
| --- | --- | --- | --- | --- | --- | --- | --- | --- | --- | --- | --- | --- | --- |
| Gene | SNP ID | | | Region | | Amino acid | Position^a^ | | Nucleotide change^b^ | | MAF^c^ | *P*^d^ | Database |
| *DEFB126* | rs11469647 | | | Exon 2 | | Q55fs^e^ | 66163..66166 | | CAAA>- | | 0.18 | 0.57 | dbSNP |
|  | rs11467417 | | | Exon 2 | | P106fs^e^ | 66314..66315 | | ->CC | | 0.41 | 0.71 | dbSNP |
| *DEFB127* | rs12480529 | | | IVS1-732bp | |  | 77454 | | T>G | | 0.32 | **0.05** | HapMap |
|  | rs2298108 | | | IVS1-61bp | |  | 78125 | | G>T | | 0.33 | 0.10 | HapMap |
|  | rs3216039 | | | IVS1-6bp | |  | 78180 | | ->C | | 0.44 | 0.23 | dbSNP |
|  | rs6055460 | | | IVS2-54bp | |  | 79362 | | A>G | | 0.34 | 0.12 | HapMap |
|  | rs12624954 | | | Exon 2 | | G31R | 79454 | | G>A | | 0.33 | 0.11 | HapMap |
| *DEFB128* | SNP2^f^ | | | IVS2-22bp | |  | 108675 | | C>A | | 0.10 | 0.86 | - |
|  | rs4813043 | | | Exon 2 | | N27K | 108728 | | A>T | | 0.36 | 0.08 | HapMap |
|  | SNP7^f^ | | | Exon 2+61bp | |  | 108990 | | T>A | | 0.49 | 0.06 | - |
| *DEFB129* | rs2298149 | | | Exon 1-41bp | |  | 147889 | | C>T | | 0.39 | 0.06 | dbSNP |
|  | rs6110460 | | | IVS1+175bp | |  | 148168 | | T>C | | 0.46 | **0.01** | HapMap |
|  | SNP6^f^ | | | Exon 2 | | L67L | 150061 | | G>A | | 0.38 | 0.11 | - |
|  | rs1053783 | | | Exon 2 | | T149S | 150306 | | C>G | | 0.07 | 0.41 | HapMap |
| *DEFB32* | rs373225 | | | IVS1+30bp | |  | 178507 | | C>A | | 0.37 | 0.25 | HapMap |
|  | rs399083 | | | IVS2-31bp | |  | 179688 | | A>G | | 0.33 | 0.16 | HapMap |
| *AK125948* | rs6053417 | | | IVS1-4234bp | |  | 5387617 | | A>G | | 0.46 | **0.05** | HapMap |
|  | rs8114316 | | | IVS2-372bp | |  | 5393914 | | T>G | | 0.49 | 0.32 | HapMap |
|  | rs1060236 | | | Exon 2, 5’UTR | |  | 5394686 | | A>G | | 0.49 | 0.26 | dbSNP |
|  | rs479233 | | | Exon 2, 5’UTR | |  | 5395074 | | A>G | | 0.34 | 0.16 | HapMap |
|  | rs6053430 | | | IVS2+4533bp | |  | 5402313 | | G>A | | 0.48 | 0.66 | HapMap |
|  | rs1977170 | | | IVS2+6220bp | |  | 5404000 | | T>G | | 0.48 | 0.64 | HapMap |
| *B. Haplotype analysis* | | | | | | | | | | | | | |
| Gene | | Haplotype^g^ | | | Frequency (%) | | | P^d^ | | 1,000,000 permutations *P* | | | |
| *DEFB126* | | H1 | [CAAA] [-] | | 42 | | | 0.95 | | 0.97 | | | |
|  |  | H2 | [CAAA] [CC] | | 38 | | | 0.94 | | 0.94 | | | |
|  |  | H3 | [-] [-] | | 20 | | | 0.93 | | 0.93 | | | |
| *DEFB127* | | H1 | TG[-]AG | | 54 | | | 0.18 | | 0.31 | | | |
|  |  | H2 | GTCGA | | 33 | | | 0.15 | | 0.17 | | | |
|  |  | H3 | TGCAG | | 11 | | | 0.59 | | 0.58 | | | |
| *DEFB128* | | H1 | CAT | | 50 | | | 0.07 | | 0.10 | | | |
|  |  | H2 | CTA | | 39 | | | 0.10 | | 0.20 | | | |
|  |  | H3 | AAA | | 8 | | | 0.87 | | 0.78 | | | |
| *DEFB129* | | H1 | CTGC | | 52 | | | 0.05 | | 0.08 | | | |
|  |  | H2 | TCAC | | 41 | | | 0.15 | | 0.24 | | | |
|  |  | H3 | CCGG | | 5 | | | 0.22 | | 0.23 | | | |
| *DEFB32* | | H1 | CA | | 61 | | | 0.29 | | 0.34 | | | |
|  |  | H2 | AG | | 35 | | | 0.12 | | 0.24 | | | |
| *AK125948* | | H1 | AGAAAG | | 43 | | | 0.31 | | 0.30 | | | |
|  |  | H2 | GTGGGT | | 34 | | | 0.09 | | 0.09 | | | |
|  |  | H3 | GTGAGT | | 11 | | | 0.48 | | 0.44 | | | |
|  |  | H4 | ATGAGT | | 8 | | | 0.06 | | 0.03 | | | |

^a^Reference sequence: NT_011387.8

^b^Most common allele is given first

^c^Minor Allele Frequency in the studied sample set

^d^FBAT association test *P*-value under the assumption of linkage

^e^Frameshift change

^f^New SNP identified at CNG

^g^SNP are in same order as in Table S2A. Only haplotypes with frequency ≥ 5% are represented
