# Supplementary material for: ADAM33, a New Candidate for Psoriasis Susceptibility
Source: PLoS One. 2007 Sep 19;2(9):e906. doi: 10.1371/journal.pone.0000906 (PMC1975467; doi:10.1371/journal.pone.0000906)
Supplement: Table S4 — Results for HLA-Cw6 tagging SNPs (PSORS1 locus). (0.09 MB DOC) [file pone.0000906.s004.doc]

Supplementary Table S4. Results for HLA-Cw6 tagging SNPs (*PSORS1* locus)

| *A. Univariate analysis* | | | | | | | | | | | | | | | | | | | | | | | |
| --- | --- | --- | --- | --- | --- | --- | --- | --- | --- | --- | --- | --- | --- | --- | --- | --- | --- | --- | --- | --- | --- | --- | --- |
| Gene | SNP | | Other name | | Amino acid | | | MAF | Set I | | | | | | Set II | | | | | | Set I + Set II | | |
|  |  |  |  |  |  |  |  |  | Nb of informative families | | Z^a^ | | P | | Nb of informative families | | Z^a^ | | *P* | | Z^a^ | *P* | |
| *HCR* | Rs130076 | | -325 C>T | | R109W | | | 0.22 | 38 | | 3.58 | | 0.0004 | | 34 | | 3.17 | | 0.001 | | 4.74 | 0.000002 | |
|  | Rs130079 | | -1723 G>T | | G575C | | | 0.24 | 38 | | 3.91 | | 0.0001 | | 34 | | 3.22 | | 0.001 | | 5.05 | <0.000001 | |
|  | Rs1576 | | -2327 C>G | | C776S | | | 0.33 | 38 | | 3.63 | | 0.0003 | | 36 | | 2.13 | | 0.03 | | 4.17 | 0.00003 | |
| *CDSN* | Rs1062470 | | 971C>T | | UTR | | | 0.37 | 39 | | 3.90 | | 0.0001 | | 41 | | 2.38 | | 0.02 | | 4.52 | 0.000006 | |
| *B. Haplotype analysis (haplotypes ≥ 2%)* | | | | | | | | | | | | | | | | | | | | | | | |
| Haplotypes^a^ | | Frequency | | Set I | | | | | | | | Set II | | | | | | | | Set I + Set II | | | |
|  |  |  |  | Nb of informative families^b^ | | Z^c^ | *P* | | | 1,000,000 permutations *P* | | Nb of informative families^b^ | | Z^c^ | | *P* | | 1,000,000 permutations *P* | | Z^c^ | | *P* | 1,000,000 permutations *P* |
| H1 CGCC | | 0.39 | | 165.8 | | (-)2.93 | 0.003 | | | 0.006 | | 66.4 | | (-)1.45 | | 0.15 | | 0.10 | | (-)3.17 | | 0.002 | 0.001 |
| H2 TTGT | | 0.22 | | 117.0 | | 4.30 | 0.00002 | | | <0.000001 | | 57.1 | | 3.84 | | 0.0001 | | 0.000004 | | 5.70 | | <0.000001 | <0.000001 |
| H3 CGCT | | 0.18 | | 91.5 | | (-)1.95 | 0.05 | | | 0.07 | | 40.6 | | (-)2.43 | | 0.01 | | 0.01 | | (-)3.06 | | 0.002 | 0.003 |
| H4 TTGC | | 0.09 | | 62.5 | | (-)0.73 | 0.47 | | | 0.41 | | 29.1 | | (-)0.99 | | 0.32 | | 0.25 | | (-)0.98 | | 0.33 | 0.27 |
| H5 CGGC | | 0.08 | | 43.0 | | (-)1.50 | 0.13 | | | 0.07 | | 12.7 | | (-)0.75 | | 0.45 | | 0.40 | | (-)1.76 | | 0.08 | 0.05 |
| H6 CTGT | | 0.02 | | 11.0 | | 0.88 | 0.38 | | | 0.72 | | 11.0 | | 0.94 | | 0.35 | | 0.40 | | 1.26 | | 0.21 | 0.36 |

^a^The 4 SNPs used for analyses are in the following order: rs130076, rs130079, rs1576, rs1062470

^b^Number of informative families estimated by FBAT

^c^Score given by FBAT
