# Supplementary material for: ADAM33, a New Candidate for Psoriasis Susceptibility
Source: PLoS One. 2007 Sep 19;2(9):e906. doi: 10.1371/journal.pone.0000906 (PMC1975467; doi:10.1371/journal.pone.0000906)
Supplement: Table S5 — Results for most significant under- and over-transmitted ADAM33 3-SNP haplotypes when stratifying according to HLA-Cw6 status in patients (for Set I). (0.07 MB DOC) [file pone.0000906.s005.doc]

Supplementary Table S5. Results for most significant under- and over-transmitted *ADAM33* 3-SNP haplotypes when stratifying according to HLA-Cw6 status in patients (for Set I)

| SNP combination^a^ | Haplotype | Frequency | Nb of informative families^b^ | Z^c^ | *P* | 1,000,000 permutations *P* |
| --- | --- | --- | --- | --- | --- | --- |
| *A. Association test performed on affected individuals carrying the HLA-Cw6 haplotype* | | | | | | |
| SNP 5/10/25 | AGT | 0.10 | 20.9 | (-)2.35 | 0.02 | 0.006 |
| SNP 5/11/23 | AGG | 0.11 | 35.9 | (-)2.64 | 0.008 | 0.009 |
| SNP 5/25/26 | ATT | 0.10 | 25.4 | (-)2.38 | 0.02 | 0.007 |
| SNP 5/26/27 | ATG | 0.09 | 21.8 | (-)1.57 | 0.12 | 0.10 |
| SNP 7/9/23 | ACC | 0.32 | 76.3 | 1.76 | 0.08 | 0.11 |
| SNP 7/16/23 | ATC | 0.32 | 70.4 | 1.70 | 0.09 | 0.16 |
| SNP 7/21/23 | AGC | 0.22 | 66.9 | 1.87 | 0.06 | 0.12 |
| SNP 15/23/24 | ACG | 0.33 | 75.9 | 2.17 | 0.03 | 0.05 |
| SNP 16/23/27 | TCG | 0.11 | 38.2 | 1.45 | 0.15 | 0.19 |
| SNP 16/26/27 | TCG | 0.10 | 33.5 | 1.99 | 0.05 | 0.07 |
| *B. Association test performed on affected individuals not carrying the HLA-Cw6 haplotype* | | | | | | |
| SNP 5/10/25 | AGT | 0.10 | 36.6 | (-)2.76 | 0.006 | 0.004 |
| SNP 5/11/23 | AGG | 0.11 | 40.9 | (-)2.19 | 0.03 | 0.02 |
| SNP 5/25/26 | ATT | 0.10 | 36.8 | (-)2.72 | 0.006 | 0.005 |
| SNP 5/26/27 | ATG | 0.09 | 33.8 | (-)2.61 | 0.009 | 0.003 |
| SNP 7/9/23 | ACC | 0.32 | 83.1 | 3.00 | 0.003 | 0.002 |
| SNP 7/16/23 | ATC | 0.32 | 84.5 | 2.98 | 0.003 | 0.002 |
| SNP 7/21/23 | AGC | 0.22 | 61.7 | 3.33 | 0.0009 | 0.0009 |
| SNP 15/23/24 | ACG | 0.33 | 81.0 | 2.50 | 0.01 | 0.01 |
| SNP 16/23/27 | TCG | 0.11 | 43.4 | 3.21 | 0.001 | 0.0009 |
| SNP 16/26/27 | TCG | 0.10 | 46.8 | 2.61 | 0.009 | 0.001 |

^a^SNP numbers refer to SNPs in Table 1.

^b^Number of informative families estimated by FBAT

^c^Score given by FBAT
